# The relationship between diabetes mellitus and 30-day readmission rates

**DOI:** 10.1186/s40842-016-0040-x

**Published:** 2017-03-22

**Authors:** Stephanie Ostling, Jennifer Wyckoff, Scott L. Ciarkowski, Chih-Wen Pai, Hae Mi Choe, Vinita Bahl, Roma Gianchandani

**Affiliations:** 1 Internal Medicine/ Metabolism, Endocrinology and Diabetes, Domino Farms Lobby G, Ste 1500, Ann Arbor, MI 48106-0482 USA; 2 6468 Robison Lane, Saline, MI 48176 USA; 3 Domino Farms Lobby G, Ste 1500, Ann Arbor, MI 48106-0482 USA; 4 University of Michigan, 300 N Ingalls, Room7A10, Ann Arbor, MI 48109-5485 USA; 5 Department of Pharmacy, University of Michigan, 1301 Catherine Street, Suite #6312 MedSci 1, Ann Arbor, MI 48109 USA; 6 Department of Surgery, University of Michigan Medical School, Taubman Center, 1500 E. Medical Center Dr., Ann Arbor, MI 48109 USA; 7 Department of Pharmacy, University of Michigan, 1111 E. Catherine St, VV 334, Ann Arbor, MI 48109 USA

## Abstract

**Background:**

It is estimated that 9.3% of the population in the United States have diabetes mellitus (DM), 28% of which are undiagnosed. The high prevalence of DM makes it a common comorbid condition in hospitalized patients. In recent years, government agencies and healthcare systems have increasingly focused on 30-day readmission rates to determine the complexity of their patient populations and to improve quality. Thirty-day readmission rates for hospitalized patients with DM are reported to be between 14.4 and 22.7%, much higher than the rate for all hospitalized patients (8.5–13.5%). The objectives of this study were to (1) determine the incidence and causes of 30-day readmission rates for patients with diabetes listed as either the primary reason for the index admission or with diabetes listed as a secondary diagnosis compared to those without DM and (2) evaluate the impact on readmission of two specialized inpatient DM services: the Hyperglycemic Intensive Insulin Program (HIIP) and Endocrine Consults (ENDO).

**Methods:**

For this study, DM was defined as any ICD-9 discharge diagnosis (principal or secondary) of 250.xx. Readmissions were defined as any unscheduled inpatient admission, emergency department (ED) visit, or observation unit stay. We analyzed two separate sets of patient data. The first pilot study was a retrospective chart review of all patients with a principle or secondary admission diagnosis of diabetes admitted to any adult service within the University of Michigan Health System (UMHS) between October 1, 2013 and December 31, 2013. We then did further uncontrolled analysis of the patients with a principal admitting diagnosis of diabetes. The second larger retrospective study included all adults discharged from UMHS between October 1, 2013 and September 30, 2014 with principal or secondary discharge diagnosis of DM (ICD-9-CM: 250.xx).

**Results:**

In the pilot study of 7763 admissions, the readmission rate was 26% for patients with DM and 22% for patients without DM. In patients with a primary diagnosis of DM on index admission, the most common cause for readmission was DM-related. In the larger study were 37,702 adult inpatient discharges between October 1, 2013 and September 30, 2014. Of these, 20.9% had DM listed as an encounter diagnosis. Rates for all encounters (inpatient, ED and Observation care) were 24.3% in patients with DM compared to 17.7% in those without DM (*p* < 0.001). The most common cause for readmission in patients with DM as a secondary diagnosis to the index admission was infection-related.

During the index hospital stay, only a small proportion of patients with DM (approximately 12%) received any DM service consult. Those who received a DM consult had a higher case mix index compared to those who did not. Despite the higher acuity, there was a lower rate of ED /observation readmission in patients followed by the DM services (6.6% HIIP or ENDO vs. 9.6% no HIIP or ENDO, *p* = 0.0012), though no difference in the inpatient readmission rates (17.6% HIIP or ENDO vs. 17.4% no HIIP or ENDO, *p* = 0.89) was noted.

**Conclusions:**

Patients with both a primary or secondary diagnosis of DM have higher readmission rates. The reasons for readmission vary; patients with a principal diagnosis of DM have more DM related readmissions and those with secondary diagnosis having more infection-related readmissions. DM services were used in a small proportion of patients and may have contributed to lower DM related ED revisits. Further prospective studies evaluating the role of these services in terms of glucose management, patient education and outpatient follow up on readmission are needed to identify interventions important to reducing readmission rates.

## Background

DM mellitus (DM) is a growing burden in the United States. It is estimated that 9.3% of the population (29.1 million people) in the United States has DM, and 28% are undiagnosed [1]. Chronic complications of DM include retinopathy, neuropathy, nephropathy, an increased risk of cardiovascular disease and major cardiac events including myocardial infarction and stroke [2]. The high prevalence of DM and its complications makes it a common comorbid condition in hospitalized patients. This leads to frequent admissions for procedures and interventions during which patients with DM are reported to have longer lengths of stay (LOS), increased hospital complications, and mortality [3–6].

In recent years, government agencies and healthcare systems are increasingly focused on 30-day readmission rates as a way to improve quality and also determine the complexity of patient populations. The Centers for Medicare and Medicaid Services (CMS) have labeled 30-day readmission rates as a measure of healthcare quality and emphasize its reduction as a strategy to reduce healthcare costs while also maintaining quality [7]. On October 1, 2012, CMS launched its Hospital Readmission Reduction Program, an item under the Patient Protection and Affordable Care Act [8]. This program determines hospital reimbursements based on five specific readmission measures: heart failure, acute myocardial infarction, pneumonia, total hip/knee arthroplasty, and COPD exacerbation [8] and hospitals with “excessive” readmissions are penalized [8].

Currently, it is estimated that 25% of all hospitalized patients have DM [3, 9, 10], but data on 30-day readmission rates for patients with DM is only just emerging. Direct medical costs due to DM were $176 billion in 2012, of which 43% was generated via inpatient care [11]. Ozieh and colleagues estimated that the United States spent approximately $218.6 billion per year in total direct health care expenditures for patients with DM and $46 billion per year in total incremental expenditures (adjusted) from 2002 to 2011, significantly higher than in those without DM [12]. The major proportion of this expense was from hospital admission and prescriptions [12]. Readmissions were a significant contribution to these expenditures. Compared to patients without DM, patients with DM were more likely to be readmitted with other comorbid conditions such as heart failure, myocardial infarction, and cardiac surgery [13]. In recent studies, the 30-day readmission rate for hospitalized patients with DM is estimated to be 14.4–22.7% [14–18], much higher than the rate for all hospitalized patients (8.5–13.5%) [19, 20].

To best address this discrepancy in readmission, it is important to determine the underlying causes of readmission in patients with DM. Some factors identified include having health care insurance [18, 21–23], the type of insurance (government vs. private or no insurance) [18, 23], male gender [18, 22, 24, 25], length of hospital stay [18, 21, 24, 26, 27], and degree of comorbidities [18, 24–26]. However, there is sparse evidence in the literature of contributing clinical factors and specific interventions to help decrease readmission rates, and therefore, strategies to resolve these factors and reduce 30-day readmissions are not commonly developed or employed.

Over the last decade, several institutions have created specialized DM teams for treatment of patients with DM, but few have reported on what percentage and which groups of hospitalized patients with DM benefit from these teams for outcomes specifically related to readmission. To evaluate the readmission rates of patients with DM at UMHS, we collected two sets of data - a three-month pilot data set followed by a one-year dataset. Baseline readmission rates in patients with a primary or secondary diagnosis of DM were evaluated to determine if there were different causes for readmission between these groups. Additionally, we also evaluated baseline characteristics of patients who were readmitted and how many of them were cared for by either of our DM services.

This project has two specific aims:

(1) Determine the incidence and causes of 30-day readmission rates for patients with a primary and secondary diagnosis of DM compared to those without DM and (2) Evaluate the impact on readmission of two specialized inpatient DM services: the Hyperglycemic Intensive Insulin Program (HIIP) and Endocrine Consults (ENDO).

## Methods

DM was defined as any medical ICD-9 discharge diagnosis (principal or secondary) of 250.xx. Encounters considered as a 30-day unscheduled readmission were either an “inpatient” admission, or an emergency department (ED) visit, or observation unit stay. ED visits and observation encounters were combined and labeled as “other” readmission encounter.

As described above, we evaluated two separate sets of patient data. The first, a pilot retrospective chart review of all patients admitted to any adult service within UMHS was conducted for a three month period, between October 1, 2013 and December 31, 2013. For the pilot data we compared readmission rates for patients with either a principal or a secondary diagnosis of DM to those without known DM. Both groups were examined for differences in rates of inpatient and emergency department /observation unit visits. Within this pilot data, we further performed extensive manual chart review and an uncontrolled analysis of patients whose primary admission diagnosis was DM related, and compared those readmitted with those not readmitted, based on age, sex, race, length of stay (LOS), discharge disposition, admitting service, discharge service (both on index), type of DM, type of DM consult (HIIP, ENDO, or no consult) Statistical analysis was performed using SPSS version 19. Results are shown as mean (SD) or percentages. T-tests were used to compare continuous variables and chi-square was used to compare categorical variables. A *p*-value of 0.05 was considered significant.

The second group of patient data was collected from a longer time frame and included inpatient adults (ages ≥ 18) who were discharged from UMHS between October 1, 2013 and September 30, 2014 with any discharge diagnosis of DM (ICD-9-CM: 250.xx). This included the subjects from the pilot group. Electronic medical records were used to gather demographic and encounter-based data. Case mix index was derived from Medicare weight per Medicare Severity Diagnosis Related Groups (MS-DRGs).

This study population was categorized based upon the type of DM consult service received during the index encounter: a) no HIIP or Endo, b) HIIP only, c) Endo only or d) both HIIP and Endo. LOS was calculated as days between the date patients arrived at the hospital, or ED arrival time if patients were admitted via the ED and their discharge date. Case mix index is based on most recent Medicare weight per MS-DRG. Bivariate analysis was carried out between various groups based on HIIP-Endo categorization. Wilcoxon rank-sum tests were conducted when comparing continuous variables; and Fisher’s exact tests were performed for categorical variables. Readmission within 30 days for both patient groups was calculated for patients who were discharged alive from the index encounter.

This study was exempt by the University of Michigan Institutional Review Board.

University of Michigan (UMHS) Specialized DM Teams.

The HIIP service is a multidisciplinary team led by a team of endocrinology faculty from UMHS and also includes four midlevel providers (Physician Assistants, Nurse Practitioners) as well as endocrine fellows. This service sees post-surgical patients who are status-post cardiac, vascular or thoracic surgery, and post-heart or lung transplant. In addition, those who are not post-surgical and have cystic fibrosis or heart failure with DM and/or hyperglycemia are also seen. This team consists of midlevel providers and endocrine faculty directly involved in the management of their patients through multiple daily patient interactions and education. Also the providers have the ability to write DM related orders for patients on this service. Post-discharge follow-up by phone and in clinic is also managed by this group for a few months until the patient is clinically stable and can go back to their primary care or endocrine providers. In contrast, ENDO is managed by the first-year endocrine fellow and UMHS clinical faculty. The ENDO team generally sees patients who are not seen by the HIIP service (e.g. general medicine, obstetrics, psychiatry, orthopedic surgery) and leaves consult recommendations with the patient’s primary team. The ENDO team does not write orders and has a narrower educational role. Post-discharge support is coordinated with the primary service.

## Results

### Three-month pilot study – focus on principal diagnosis of DM

There were a total of 7763 admissions between 10/1/2013 and 12/31/2013 of which, 97.3% (7554) were discharged alive and 25% had a diagnosis of DM. Patients with any diagnosis of DM on index admission had higher overall 30 day readmission rates (Inpatient plus other) of 26.4% compared to patients without DM of 22.6% (*p* < 0.001). Inpatient readmission rates were significantly higher amongst patients with either a primary or a secondary diagnosis of DM (344/1940 or 18%) than those without DM (775/5823 or 13.7%). Patients with a primary, secondary, or no known diagnosis of DM had readmission rates of 40.5, 25.8 and 22.5%, respectively. Those with a primary diagnosis of DM had the highest overall readmission rate, significantly greater for both inpatient and ED/observation unit encounters (*p* < 0.001 and 0.02) respectively (Table 1).

**Table 1:** Pilot study data – readmission rates by DM diagnosis for patients with index admission between 10/1/2013 and 12/31/2013

	Principal Dx of DM (n)	Secondary Dx of DM (n)	*p*-value	Any DM	No DM Dx (*n*)	*p*-value
Index discharge *N* = 7763	121	1819		1940	5823	
Discharged alive *N* = 7554	121	1757		1878	5676	
30 day Readmissions^a^	*n* (%)	*n* (%)			*n* (%)	
Inpatient readmission	31(25.6)	313(17.8)	0.02‡	344(18)	775(13.7)	<0.001‡†#
Other encounters	18(14.9)	141(8)	0.005‡	159(8.4)	504(8.9)	0.02^b^ 0.23^c^
Total	49(40.5)	454(25.8)		503(26.8)	1279(22.5)	
Endocrine HIIP service	34(28)	275(15)		309(16)	97(1.7)	<0.001

^a^Among patients discharged alive; ^b^Comparison made to primary diagnosis of DM group; ^c^Comparison made to secondary diagnosis of DM; #any DM vs. no DM

We further evaluated the group of patients with a primary diagnosis of DM (*n* = 121) (Table 2, Appendix). Their mean age was 50 years, and 67% had acute or chronic renal disease. 30% were admitted for a surgical procedure, and the majority (95%) received insulin while inpatient. Of those who had a recent hemoglobin A1c test, the majority were uncontrolled as defined by a HbA1c > 7.0% (74.4%). Only 28% of patients with a primary diagnosis of DM were seen by either the HIIP or ENDO service for DM management. 26% (31/121) of patients with a primary diagnosis of DM were readmitted and the major cause for readmission was DM-related (Table 3).

**Table 2:** Baseline characteristics of patients admitted with a primary diagnosis of DM

*N* = 121	Mean	%
Age ( years)	49.7	
Inpatient LOS (days)	4.7	
Female	54	44.6
With renal disease (acute or chronic)	81	66.9
HbA1c performed within 2 months prior to index encounter	43	35.5
HbA1c > 7.0% (among patients with HbA1c)	32	74.4
HbA1c > 10.0%	13	30.2
During index encounter		
Undergoing surgical procedure	37	30.6
Receiving insulin	115	95.0
Endocrine (HIIP/Endo) consult^a^/office visit		
During index encounter	34	28.1
Endocrine post-discharge appointment scheduled	31	25.6
Endocrine office visit billed	8	6.6

^a^Endocrine consult here refers to either a HIIP consult or Endocrine consult; Note: *n* = 121 refers to the number of encounters, not the number of individuals, some individuals had more than one readmission

**Table 3:** Principal diagnosis for inpatient readmission encounters among patients with principal diagnosis of DM on index admission in pilot study

*n* = 31	
DM = 35.4%	
Infection = 19.4%	
Renal disease = 19.4%	
Other = 25.8%	

The demographic distribution of the 121 encounters with a primary diagnosis of DM is described in Table 4 by readmission status (readmitted vs. not-readmitted). The two groups were similar for age, sex, race, type of DM, and length of stay. On univariate analysis, there were no statistically significant differences between the readmitted and not readmitted groups for admitting or discharging service, insurer, or discharge disposition.

**Table 4:** Demographic description at index encounter based on readmission status (*n* = 121)

	Readmitted (44)	Not-readmitted (77)	*P*-value
Age(y)Mean ± S.D	49.3 ± 15	50 ± 18	0.85
Sex (M/F)	24/20	43/34	0.89
Race (Black/non-black)	15/29	23/54	0.63
DM (T1/T2)	20/24	32/45	0.67
Length of stay	5.8 ± 6	4.1 ± 4	0.09

### Twelve-month study- focus on secondary diagnosis of DM

In our larger data set there were 37,702 adult inpatient discharges between October 1, 2013 and September 30, 2014 and 21% (7872) of patients had known DM (Table 5). Readmission rates for all encounters (inpatient, ED and Observation care) were 24.5% in patients with DM and 17.7% in those without DM (*p* < 0.001). The 30-day readmissions for DM for an inpatient and other (ED plus observation unit), were 17.5 and 9.3%, respectively. Patients with DM were significantly more likely to be readmitted compared to patients without DM for all three encounter types (*p* < 0.001). During the index hospital stay, 12% of patients with DM received a DM service consult; (9.6% by HIIP and 3.2% by ENDO) (Table 5).

**Table 5:** Unscheduled readmissions by type of DM consult received

		Unscheduled Readmission					
Consult type	Index discharge	Inpatient encounter		Other encounter		Any encounter	
		*N*%	*p*-value	*n*%	*p*-value	*n*%	*p*-value
All Inpatient adult discharges	36,820	4798 (13)	-	2908(7.9)	-	7009(19)	-
Inpatient adult discharges without DM	29,215 (79.4)	3468(11.9)	<0.001*	2204 (7.5)	<0.001*	5163(17.7)	<0.001*
Inpatient adult discharge with DM	7605 (20.6)	1330(17.5)	-	704 (9.3)	-	1846(24.3)	-
No HIIP/Endo	6559	1142(17.4)	--	632(9.6)	--	1606(24.5)	--
Either HIIP/Endo	1046	184(17.6)	0.8953	69(6.6)	0.0012	234(22.4)	0.1398
HIIP only	728	127(17.4)	1.000	41(5.6)	0.0003	159 (21.8)	0.1211
Endo only	243	36(14.8)	0.342	19(7.8)	0.436	46(18.9)	0.0477

All *p*-values represent comparison of the readmission rate for patients who received no HIIP or endocrine consult to other consult categories; *These *p*-values compare the overall readmission rate for each encounter type (shaded row) to the readmission rate for inpatient adult discharge without DM

Amongst the readmitted patients, a similar proportion of 12% (243/1846) had received a DM service consult during index admission. There was a lower rate of ED /observation readmission rate in patients followed by the DM services, the bulk of contribution being from HIIP (*p* = 0.0012). There was no difference in the inpatient readmission rates in patients with a DM consult vs. those without (*p* = 0.8953). The 30-day all-cause inpatient unscheduled readmission rate was 17.4% for HIIP group, compared to 14.8% for ENDO group (*p* = 0.3733).

The demographic characteristics of adults with DM who were discharged between October 1, 2013 and September 30, 2014, separated by the consult received, are provided in Table 6. Of the 7605 DM patients discharged alive, the average age was 62.7 years old, 56% were male and 48% had acute or chronic renal insufficiency. The mean LOS for all patients was 6.5 days. Only a small percentage (13.7%) of patients received a DM-related consult.

**Table 6:** Demographic information of patients with DM

	Total (*n*) *N*(%)	No HIIP, No ENDO	HIIP only	ENDO only	HIIP or ENDO	Both HIIP and ENDO
Discharged	7872	6804	745	245	1068	78
Discharged alive	7605 (96.6%)	6559 (96.4%)	728 (97.7%)	243 (99.2%)	1046 (97.9%)	75 (96.2%)
Among discharged alive						
Avg age	62.7	63.2	61.6	53.1	59.3	57.1
Race -black	1206 (15)	1076 (16.4)	87 (12)	33 (13.6%)	130 (12.4%)	10 13.3%
Gender – M	4231 (55.6)	3582 (54.6)	470(65.6)	133 (54.7%)	649 (62%)	46 61.3%
Renal Ds.	3719 (48.)	3153 (48.1)	400 (54.9)	118 (48.6%)	566(54.1)	48 (64.0%
Mean LOS-days	6.5	5.8	11.1	7.8	10.9	19.3
Case mix index	2.10	1.84	4.12	1.99	3.73	5.54
Median Case mix index	1.47	1.35	2.82	1.54	2.28	2.39
Race – black	1.30	1.22	1.92	1.12	1.54	0.83
Race – non-black	1.51	1.38	3.00	1.56	2.55	3.15

The average LOS for all patients was 6.5 days and average CMI was 2.10. When the DM groups were separated by service received, the shortest LOS was among patients who did not receive HIIP or ENDO services (5.8 days) as was the lowest CMI at 1.84. . Patients who received HIIP services only had an average LOS of 11.1 days, ENDO only 7.8 days. The longest average LOS among those who received both HIIP and ENDO services (19.3 days), and the average CMI of this group was 5.54 which means that this complex patient group which transferred through many different services.

Again the increased clinical severity of the patient population serviced by HIIP and the combined group is reflected in the higher CMI and LOS. Despite the complexity of this group, they still had a lower rate of ED/observation readmissions.

The median LOS and CMI of patients based on whether and type of DM consult they received is shown in Table 7. Patients who did not receive HIIP or ENDO had significantly shorter median LOS (4 vs. 7 days respectively, *p* < 0.0001) and CMI than those who received HIIP or ENDO consult ENDO (1.35 vs. 2.28 respectively, *p* < 0001), (Table 6).

**Table 7:** Comparison of inpatient length of stay and case mix index to ‘No HIIP, No ENDO’

	Total	No HIIP, No ENDO	HIIP only	*P*-value	ENDO only	*P*-value	HIIP or ENDO	*P*-value
Median Inpatient LOS	4	4	7	<0.0001	5	<0.0001	7	<0.0001
Median Case Mix Index	1.47	1.35	2.82	<0.0001	1.54	<0.0001	2.28	<0.0001

We also evaluated the principal reasons for readmission in this large cohort (Table 8). The most common causes included infections (septicemia, postoperative infections, urinary tract infections, pneumonia, Clostridium difficile, and venous line infections), acute renal failure, complications of transplant, heart failure exacerbation, myocardial infarctions, and DM-related complications.

**Table 8:** Principal discharge diagnosis of inpatient readmission encounter in patients with any diagnosis of DM in the larger study

Count	Description
203 (38%)	Infection total
69 (13%)	Heart failure
55 (10%)	Transplant complication
48 (9%)	Kidney failure
45 (8%)	Dm
115 (21%)	Miscellaneous (including DVT and GI bleed)
535	Total

## Discussion

The prevalence of DM continues to increase in the United States and presents a growing problem for health care [1]. It is a significant financial burden for patients, health care providers and society. Nearly a quarter of hospitalized patients have DM, leading to increased mortality, morbidity, and hospital complications [3]. Patient with DM have higher early readmission rates compared to the general population. Thirty-day readmission rates are a key quality indicator. We therefore chose to evaluate the prevalence of DM in our hospital population and the frequency and reasons for readmissions. Understanding the factors associated with early readmission in patients with DM will enable the development and implementation of strategies to reduce readmission rates in this high risk population. This study contributes to the limited body of literature on 30-day readmission rates in patients with DM and determines the incidence and causes of 30-day readmissions for patients with a primary or secondary diagnosis of DM and evaluates the impact on readmission of specialized inpatient DM services.

Twenty five percent of admitted patients had a primary or secondary diagnosis of diabetes in our pilot study and twenty one percent in our larger study. These rates are consistent with the literature [5]. Patients with either a primary or secondary diagnosis of DM were significantly more likely to be seen within 30 days of discharge compared to patients without DM for all three encounter types: inpatient admission, observation admission and ED visit. Patients with a primary, secondary, or no known diagnosis of DM had readmission rates of 40.5, 25.8 and 22.5%, respectively. This very high readmission rate in patients with an index admission for diabetes highlights the importance of improving our understanding of the reasons for readmission in patients admitted with a principal diagnosis of diabetes.

In our pilot study we found that in patients with a principal diagnosis of DM, most required insulin, roughly two-thirds had renal disease and almost a third had very poorly controlled diabetes with an A1C > 10%. Despite this only 28% had a DM related consult at index encounter and only 25% had a follow up appointment scheduled for DM- related service. Only 6.6% actually followed up. Since diabetes was the principal reason for readmission in 35.4% of readmission for patients with a principal diagnosis of diabetes on index admission, the involvement of DM related services in the hospital and on discharge may have reduced the readmission outcome.

In the larger study where we evaluated patients with secondary diagnosis of DM, infections were the overwhelming cause of readmission. Multiple studies have shown that diabetes is a significant risk factor for infection [28]. Furthermore, good glycemic control perioperatively has been shown to reduce surgical site infections [29]. However, we were unable to determine the relative contribution of various risk factors such as glycemic control, immunosuppression and infections in this analysis. Since we did not evaluate hospital glucose control, the effect of glycemic management by DM services on readmission is not possible to evaluate. Twelve percent of patients with DM as a secondary diagnosis received either a HIIP or ENDO consult during the index admission. There were no significant differences between type of inpatient diabetes consult on inpatient admissions. However, patients who received either a HIIP or ENDO consult, or those who received only a HIIP consult, were significantly less likely to have an emergency department visit or observation unit stay. Patients with a HIIP consult had higher complexity with significantly higher case mix index than those with either an ENDO or no consult (4.12 for HIIP vs. 1.99 for ENDO and 1.84 for no consult). They also had longer LOS reflecting their case complexity and comorbidities (11.0 for HIIP vs. 7.6 or ENDO and 5.6 for no consult). In assessing these data it is important to consider that the patients populations based on type of DM consult (HIIP vs ENDO vs neither) varied immensely and we could not control all these factors for outcomes. In addition there were several other limitations in our study. It is a retrospective review and data are derived from the data warehouse and chart review. Patients without a billing diagnosis of DM who had known DM would not be included and therefore we may not have captured all patients with DM. While there were some direct comparisons between HIIP only and endocrine only groups, it is important to note that these two consult groups service very different patient populations. Finally, data that was collected represents only a single, academic institution and may not be representative of patients with DM at other institutions. This is one of the first studies evaluating DM readmissions separated by a primary and secondary diagnosis of DM and finding that they have separate reasons for readmissions.

Future prospective controlled studies evaluating readmissions in DM patients and controlling for glycemic management, other comorbidities and managed with and without a DM service may help determine other modifiable risk factors associated with early readmission. Identifying and understanding several of these factors will help devise DM services to tailor care to hospital glucose control and beyond into education and close post-discharge follow-up with the intention of reducing early readmission.

## Conclusion

Patients with a primary and secondary diagnosis of DM have higher readmission rates than patients without known DM. Reasons for readmission varied, those with a principal admitting diagnosis having more readmissions with diabetes related issues while those with a secondary admitting diagnosis of diabetes having more infection-related readmissions. DM services were utilized in very small proportion of patients with DM and may have contributed to lower ED revisits by providing more robust diabetes management, discharge planning and instruction. Their impact on readmission in prospective studies needs to be evaluated.

## Appendix

Appendix xx. Diagnosis codes for renal diseases (from Leapfrog – VB 2011)

403.00, 403.01, 403.10, 403.11, 403.90, 403.91

404.00–404.03

404.10–404.13

404.90–404.93

582.xx–587.xx

753.14

## Abbreviations

CMI: Case mix index
CMS: Centers for Medicare and Medicaid Services
DM: Diabetes mellitus
DVT: Deep vein thrombosis
DX: Diagnosis
ED: Emergency department
ENDO: Endocrine Consult Service
GI: Gastrointestinal
HIIP: Hyperglycemic Intensive Insulin Program
LOS: Length of stay
MS-DRGs: Medicare Severity Diagnosis Related Groups
UMHS: University of Michigan Health System
